# Effect of anlotinib as a third‐ or further‐line therapy in advanced non‐small cell lung cancer patients with different histologic types: Subgroup analysis in the ALTER0303 trial

**DOI:** 10.1002/cam4.2913

**Published:** 2020-02-16

**Authors:** Ying Cheng, Baohui Han, Kai Li, Qiming Wang, Li Zhang, Jianhua Shi, Zhehai Wang, Jianxing He, Yuankai Shi, Weiqiang Chen, Xiuwen Wang, Yi Luo, Kejun Nan, Faguang Jin, Baolan Li, Jing Zhu

**Affiliations:** ^1^ Jilin Cancer Hospital Changchun China; ^2^ Shanghai Chest Hospital Shanghai Jiaotong University Shanghai China; ^3^ Tianjin Medical University Cancer Hospital Tianjin China; ^4^ Affiliated Cancer Hospital of Zhengzhou University Henan Cancer Hospital Zhengzhou China; ^5^ Peking Union Medical College Hospital Beijing China; ^6^ Lin Yi Cancer Hospital Linyi China; ^7^ Shandong Cancer Hospital Jinan China; ^8^ The First Affiliated Hospital of Guangzhou Medical University Guangzhou China; ^9^ Cancer Hospital Chinese Academy of Medical Sciences Beijing China; ^10^ Lanzhou Military General Hospital Lanzhou China; ^11^ Qilu Hospital of Shandong University Jinan China; ^12^ Hunan Cancer Hospital Changsha China; ^13^ The First Affiliated Hospital of Xi'An Jiaotong University Xi'an China; ^14^ Tang Du Hospital Xi'an China; ^15^ Beijing Chest Hospital Capital Medical University Beijing China; ^16^ Jilin Cancer Hospital Jilin China

**Keywords:** ACC, anlotinib, antiangiogenesis, SCC, third‐line

## Abstract

**Background:**

Anlotinib showed significant survival benefits in advanced non‐small cell lung cancer (NSCLC) patients as a third‐ or further‐line treatment in the ALTER0303 trial. We aimed to evaluate the efficacy of anlotinib in patients with different histologies.

**Methods:**

The ALTER0303 trial was a randomized, open‐label, phase 3 study of anlotinib in NSCLC patients previously treated with at least two lines of chemotherapy or a tyrosine kinase inhibitor (TKI) in 31 centers in China. Patients were randomly assigned at a 2:1 ratio to receive anlotinib (12 mg QD from days 1 to 14 of a 21‐day cycle) or placebo until progression or intolerable toxicity. The primary endpoint was overall survival (OS). We assessed the efficacy of anlotinib in histological subgroups in the full analysis set.

**Results:**

In the ALTER0303 trial, 336 patients had the histological subtype of adenocarcinoma (ACC), 86 patients had the histological subtype of squamous cell carcinoma (SCC), and 15 patients had another subtype. In the ACC subgroup, the median OS time was significantly improved with anlotinib compared with placebo (9.6 months vs 6.9 months, *P* = .0051), as was the median progression‐free survival (PFS) time (5.5 months vs 1.4 months, *P* < .0001). In the SCC subgroup, the median OS time was 10.7 months in the anlotinib group and 6.5 months in the placebo group (*P* = .2570), and the median PFS time was 4.8 months and 2.7 months (*P* = .0004), respectively. The common adverse events observed in the SCC and ACC subgroups were similar.

**Conclusions:**

Our findings suggest that anlotinib significantly improves PFS and OS in ACC patients and has a tendency to prolong survival in SCC patients.

## Graphical abstract

In this study, we evaluated the efficacy of anlotinib as third‐ or further‐line treatment for patient with different histological types of NSCLC. In the ALTER0303 trial, 336 patients had the histological subtype of adenocarcinoma (ACC), 86 patients had the histological subtype of squamous cell carcinoma (SCC), and 15 patients had another subtype. Our findings suggest that anlotinib significantly improves PFS and OS in ACC patients and has a tendency to prolong survival in SCC patients.

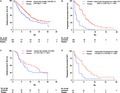

## 1 INTRODUCTION

During recent years, histological types and gene mutation types have become the basis of treatment for non‐small cell lung cancer (NSCLC).^1^, ^2^, ^3^, ^4^ Differentiation between squamous cell carcinoma (SCC) and nonsquamous tissues is critical for determining treatment strategies for patients with NSCLC. For example, pemetrexed, which was approved in 2004 for advanced NSCLC independent of the histological type^5^ and was subsequently revised for second‐line indications, is now an option only for adenocarcinoma (ACC) patients.^2^ The development of epidermal growth factor receptor tyrosine kinase inhibitors (EGFR TKIs) has progressed significantly, and many novel EGFR TKIs have been developed. Guidelines suggest that patients without sensitizing EGFR mutations not be treated with EGFR TKIs in any line of therapy.^6^ Most patients with sensitizing EGFR mutations are nonsmokers or former light smokers with ACC.^7^ Therefore, EGFR TKIs perform well in ACC patients, but few provide clinical benefits for SCC patients. Immunotherapy has made a breakthrough. Nivolumab and pembrolizumab have shown survival benefits in both ACC and SCC patients as second‐line or subsequent therapies,^8^, ^9^, ^10^ but they are difficult to apply widely in China because of economic reasons.

Angiogenesis is very important for several aspects of tumor development, including tumor growth, invasion, and metastasis.^11^ Therefore, antiangiogenesis therapy plays an important role in oncotherapy. However, bevacizumab was approved only for nonsquamous NSCLC.^12^ Nintedanib prolonged overall survival (OS) only in patients with ACC.^13^ Sorafenib improved only the progression‐free survival (PFS) of NSCLC patients,^14^ while sunitinib and pazopanib showed no clear efficacy.^15^, ^16^, ^17^ Anlotinib is a multitargeted TKI that inhibits VEGFR, PDGFR, FGFR, c‐Kit, and other kinases.^18^ The ALTER0303 trial, which was a randomized, open‐label, phase 3 trial (NCT02388919), reported the efficacy of anlotinib for the third‐ and further‐line treatment of advanced NSCLC. Anlotinib significantly prolonged OS (9.6 months vs 6.3 months) and PFS (5.4 months vs 1.4 months).^19^ These results suggest that anlotinib is a promising treatment for advanced NSCLC, and it has been approved by the China Food and Drug Administration (CFDA) for this population. However, its efficacy in different histological subtypes of NSCLC is not yet clear.

Here, we report a subgroup analysis that was performed to assess the efficacy of anlotinib in subgroups of patients with different histological subtypes and who received different treatments and treatment durations in the ALTER‐0303 trial.

## 2 METHODS

### 2.1 Study design and participants

The ALTER0303 trial was a multicenter, double‐blind, randomized, phase III trial that enrolled patients from 31 centers in China. The design and results of the ALTER0303 trial were published previously. Briefly, eligibility criteria were as follows: patients aged 18‐75 years with metastatic or recurrent NSCLC confirmed by histology or cytology; patients with driver mutations (EGFR mutation or ALK rearrangement) needed to progress from at least one line of chemotherapy and one line of TKI therapy, while patients without driver mutations needed to progress after at least 2 lines of chemotherapy. Key exclusion criteria included patients with centrally located SCC with cavitary features and patients with brain metastases that were uncontrolled and/or controlled for <2 months. The predefined stratification factors were as follows: histopathological classification (ACC vs SCC vs others), number of metastases (≤3 vs >3), and driver gene (EGFR or ALK) mutation status (positive vs negative).

### 2.2 Treatments

Oral anlotinib (12 mg/day) or matched placebo was administered. One cycle was defined as 2 weeks on treatment followed by 1 week off treatment. Treatment was continued until disease progression or intolerance to adverse reactions. Dose modifications (10 mg/day or 8 mg/day) of anlotinib were allowed according to the degree of drug‐related toxicity (according to NCI CTC AE 4.0) and the potential benefit to the patient. Briefly, if the patient with 12 mg/day presented ≥grade 3 adverse events (AE), the dose was reduced to 10 mg/day or 8 mg/day. If the dose of 8 mg/day was not tolerated, treatment was terminated. According to the Response Evaluation Criteria in Solid Tumors (RECIST) version 1.1, tumor assessment was performed within 2 weeks before treatment with computed tomography. After treatment initiation, tumors were evaluated once every cycle during the first two cycles and then once every two cycles, and patients were followed up every eight weeks to assess clinical outcomes, including toxicity, efficacy, and survival, until patients died or until the day of data cut‐off was reached (January 6th, 2017).

### 2.3 Outcomes

The primary endpoint was OS. The secondary endpoints included PFS, the objective response rate (ORR), the disease control rate (DCR), and quality of life (QoL). Tumor response and progression were assessed according to the RECIST version 1.1. The safety of the treatment was assessed by AEs, and the severity of AEs was graded according to the NCI Common Terminology Criteria for Adverse Events version 4.02.

### 2.4 Statistical analysis

In this subgroup analysis, patients were divided into two subgroups according to histological type: SCC and ACC. In these subgroups, we analyzed the efficacy of anlotinib in patients treated with different front‐line therapies and different treatment durations. Effect outcomes were analyzed based on the full analysis set, which was defined as all patients treated with the study drug at least once in accordance with the intend‐to‐treat principle. Safety outcomes were analyzed in the safety set, which included all randomized patients who received at least one dose of the study medication and had records of safety. Demographic data, outcome data, and other clinical parameters are presented as the proportions for categorical variables and as the mean ± SD for continuous variables. The ORR and DCR were compared using the χ^2^ test or Fisher's exact test, as appropriate. Continuous variables were tested using an independent samples *t* test. The Kaplan‐Meier method was used to estimate the survival curves for OS and PFS. Differences in survival were assessed using the log‐rank test. The proportional hazards (Cox) model was used to estimate hazard ratios (HRs). Two‐sided *P* values <.05 were considered statistically significant. All analyses were performed using SAS software version 9.4.

## 3 RESULTS

### 3.1 Patients and treatment

In total, 437 NSCLC patients were enrolled. Among them, 336 patients had the histological subtype of ACC, 86 patients had the histological subtype of SCC, and 15 patients had another histological subtype. Patient characteristics are summarized in Table 1. In total, 38.09% of ACC patients and 9.30% of SCC patients had an EGFR mutation, and 60.12% of ACC patients and 24.42% of SCC patients had previously received targeted therapy. In ACC patients, the most common chemotherapy agents were pemetrexed, docetaxel, and gemcitabine, and the most common targeted agents were gefitinib, erlotinib, and icotinib. After progression, 27 and 13 ACC patients in the anlotinib and placebo groups, respectively, received targeted agents, and 26 and 12 ACC patients, respectively, received chemotherapy agents. In SCC patients, the most common chemotherapy agents were docetaxel, gemcitabine, and paclitaxel. A few SCC patients received gefitinib (n = 7), erlotinib (n = 3), icotinib (n = 3), or crizotinib (n = 4). After progression, 3 and 3 SCC patients in the anlotinib and placebo groups, respectively, received targeted agents, and 6 and 2 SCC patients, respectively, received chemotherapy agents.

**Table 1:** Baseline characteristics of patients in the adenocarcinoma (ACC) and squamous cell carcinoma (SCC) subgroups

Characteristic	ACC	SCC
Age		
≤60	200 (59.52%)	38 (44.19%)
61‐69	115 (34.23%)	41 (47.67%)
≥70	21 (6.25%)	7 (8.14%)
Sex		
Male	194 (57.74%)	78 (90.70%)
Female	142 (42.26%)	8 (9.30%)
EGFR		
Negative	208 (61.91%)	78 (90.70%)
Positive	128 (38.09%)	8 (9.30%)
Clinical stage		
Other	0	2 (2.33%)
IIIB	9 (2.68%)	11 (12.79%)
IV	327 (97.32%)	73 (84.88%)
Number of metastases		
>3	154 (45.83%)	26 (30.23%)
≤3	182 (54.17%)	60 (69.77%)
*ALK* rearrangement		
Deficiency	3 (0.89%)	1 (1.16%)
Negative	328 (97.62%)	83 (96.51%)
Positive	5 (1.49%)	2 (2.33%)
Chemotherapy		
Second line	186 (55.36%)	49 (56.98%)
Third line	147 (43.75%)	36 (41.86%)
First line	3 (0.89%)	1 (1.16%)
Targeted therapy		
No	134 (39.88%)	65 (75.58%)
Yes	202 (60.12%)	21 (24.42%)
Radiotherapy		
No	204 (60.71%)	40 (46.51%)
Yes	132 (39.29%)	46 (53.49%)
ECOG PS score		
0	67 (19.94%)	13 (15.12%)
1	267 (79.46%)	72 (83.72%)
2	2 (0.60%)	1 (1.16%)
Smoking history		
Current/former	148 (44.04%)	62 (72.09%)
Never	188 (55.95%)	24 (27.91%)

### 3.2 Efficacy of anlotinib for ACC and SCC

**Figure 1:**
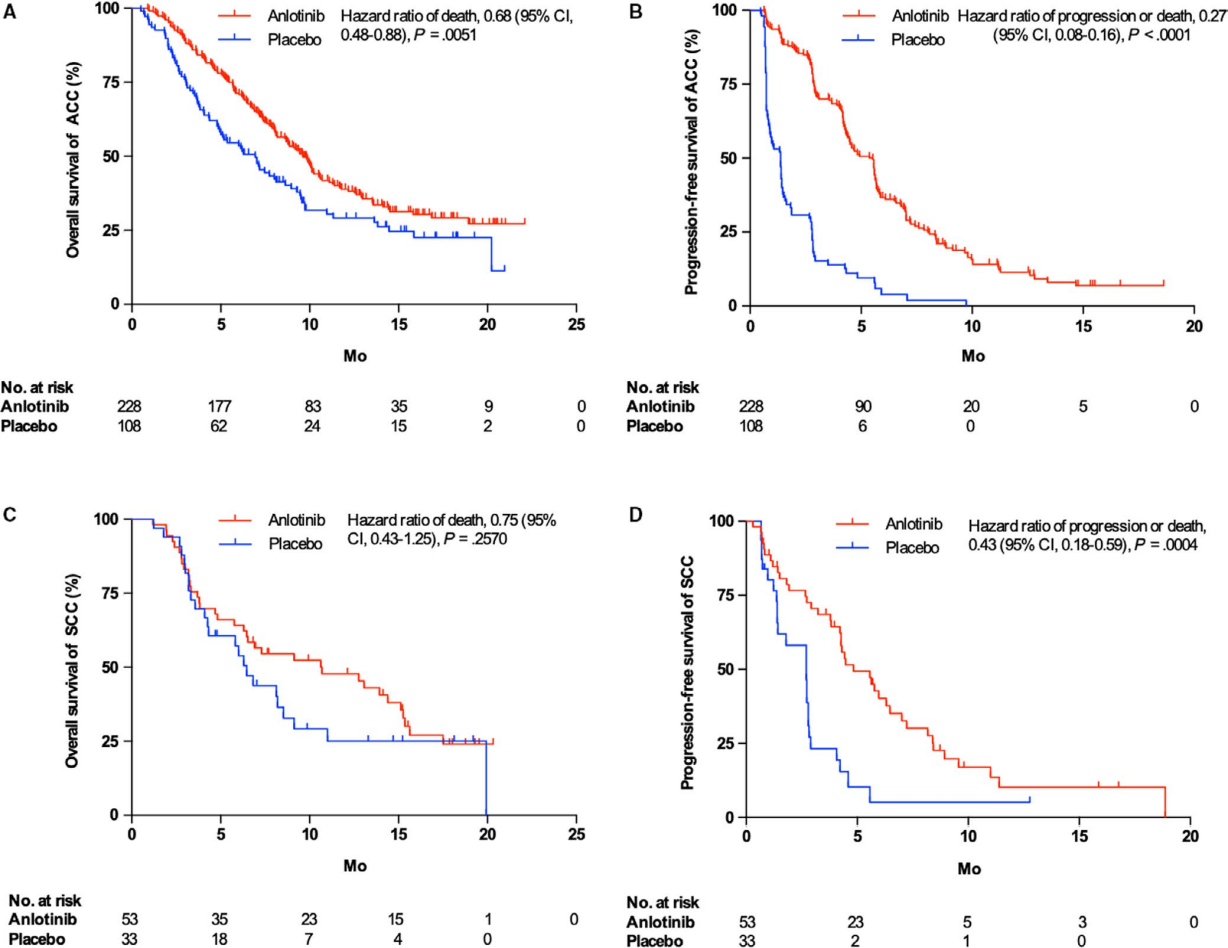
Overall survival (A) and progression‐free survival (B) in adenocarcinoma patients; overall survival (C) and progression‐free survival (D) in squamous cell carcinoma patients

For patients with the ACC subtype, the median duration of follow‐up was 7.6 months. In total, 142 of 228 patients in the anlotinib group and 77 of 108 patients in the placebo group died, and the median treatment cycle was 6 in the anlotinib group and 2 in the placebo group. The median OS time was 9.6 months in the anlotinib group (n = 228) and 6.9 months in the placebo group (n = 108) (*P* = .0051), and the median PFS time was 5.5 months and 1.4 months, respectively (*P* < .0001) (Figure 1A,B). For patients with the SCC subtype, the median duration of follow‐up was 6.9 months. Thirty‐six of 53 patients in the anlotinib group and 24 of 33 patients in the placebo group died, and the median treatment cycle was 7 in the anlotinib group and 2 in the placebo group. The median OS time was 10.7 months in the anlotinib group (n = 53) and 6.5 months in the placebo group (n = 33) (*P* = .2570) (Figure 1C), and the median PFS time was 4.8 months and 2.7 months, respectively (*P* = .0004, Figure 1D). The DCR (82.89% vs 33.33%, *P* < .0001) and ORR (9.65% vs 0.93%, *P* = .002) significantly improved with anlotinib treatment in ACC patients. However, there were no significant differences in the DCR (71.70% vs 51.51%, *P* = .0580) or ORR (7.55% vs 0%, *P* = .2758) between SCC patients in the anlotinib and placebo groups (Table S1). The subsequent treatment of ACC and SCC patients are shown in Table S2.

### 3.3 Subgroup analysis in ACC and SCC

**Figure 2:**
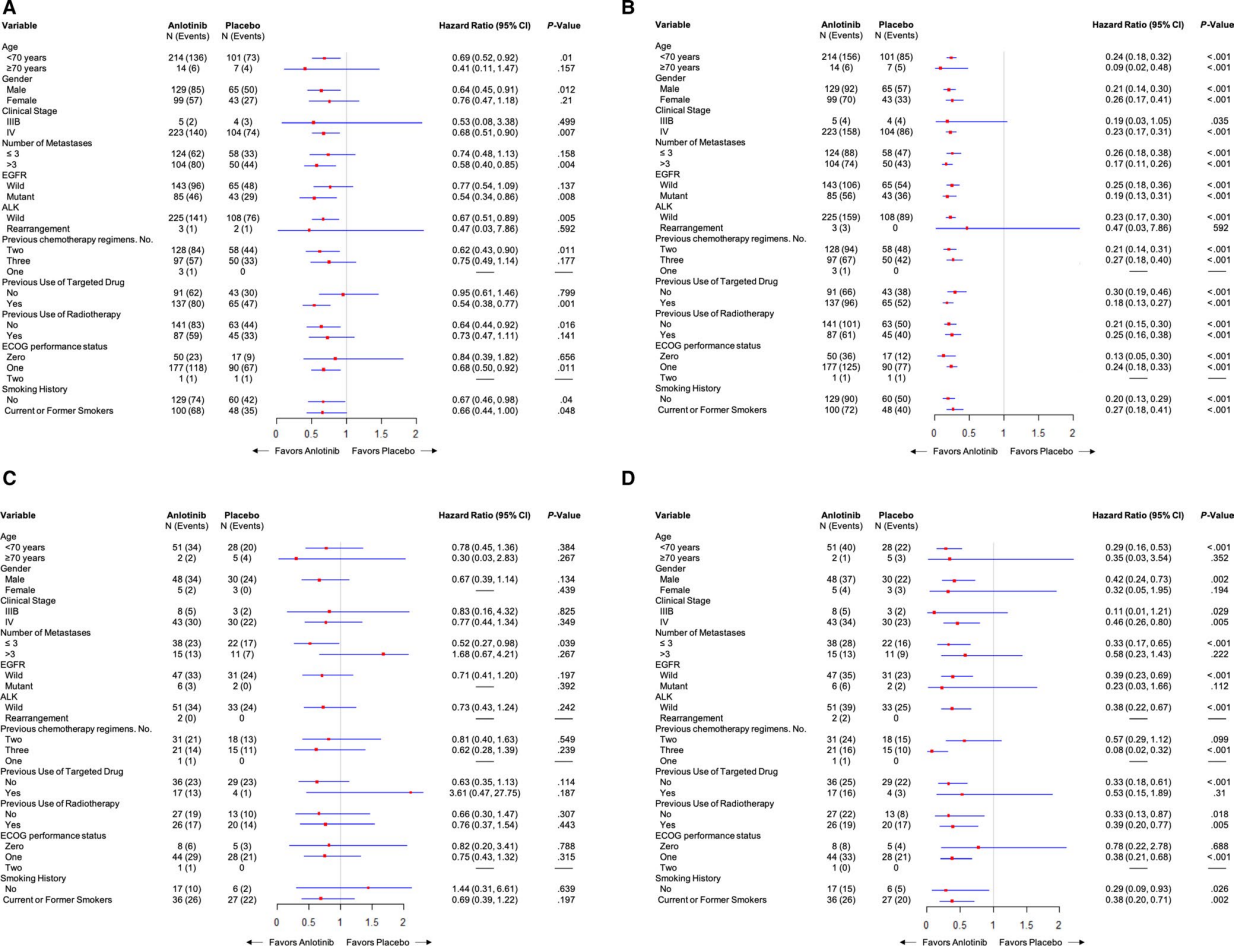
Subgroup analysis of overall survival (A) and progression‐free survival (B) in adenocarcinoma patients; subgroup analysis of overall survival (C) and progression‐free survival (D) in squamous cell carcinoma patients

In ACC patients, anlotinib significantly prolonged PFS in most subgroups (Figure 2A). OS was significantly prolonged with anlotinib in patients with an EGFR mutation, an Eastern Cooperative Oncology Group performance status (ECOG PS) score of 1, and >3 metastases and in patients who received two chemotherapy regimens or targeted regimens (Figure 2B). In SCC patients, PFS was significantly prolonged with anlotinib in subgroups with large sample sizes, and OS was significantly improved in patients with ≤3 metastases (Figure 2C,D).

### 3.4 Safety of anlotinib in ACC and SCC

In this study, we also evaluated the safety of anlotinib for the treatment of patients in the ACC and SCC subgroups. AEs (grades 1‐5) observed in these groups are summarized in Table 2. In the anlotinib group, 20 ACC patients had dose reductions, while 35 ACC patients discontinued treatment because of AEs. In the placebo group, eight ACC patients discontinued treatment because of AEs. In ACC patients treated with anlotinib, the most common AEs were hypertension (65.79%), an elevated thyroid‐stimulating hormone (TSH) level (45.61%), fatigue (45.61%) and a hand and foot skin reaction (HFSR, 44.74%), and the most common grade 3 or worse AEs were hypertension (11.84%), an HFSR (3.51%), hyponatremia (3.07%), and lymphocyte reduction (3.07%). In the anlotinib group, four SCC patients had dose reductions, and eight SCC patients discontinued treatment because of AEs. In placebo group, four SCC patients discontinued treatment because of AEs. The AEs of the SCC subgroup were similar to those of the ACC subgroup, and the most common AEs were hypertension (64.15%), fatigue (47.17%), an elevated TSH level (39.62%), and anorexia (39.62%), and the most common grade 3 or worse AEs were hypertension (18.87%), hyponatremia (11.32%), hemoptysis (9.43%), and prolongation of the QT interval (5.66%).

**Table 2:** Common adverse events (≥10%) in ACC and SCC patients

Adverse event	All grades	≥Grade 3	All grades	≥Grade 3
ACC	Anlotinib, n = 228		Placebo, n = 108	
Hypertension	150 (65.79%)	27 (11.84%)	13 (12.04%)	0 (0%)
Fatigue	104 (45.61%)	0 (0%)	29 (26.85%)	0 (0%)
TSH elevation	104 (45.61%)	1 (0.44%)	4 (3.7%)	0 (0%)
HFSR	102 (44.74%)	8 (3.51%)	10 (9.26%)	0 (0%)
Triglyceride elevation	91 (39.91%)	6 (2.63%)	21 (19.44%)	0 (0%)
Anorexia	88 (38.6%)	2 (0.88%)	24 (22.22%)	1 (0.93%)
TC elevation	83 (36.4%)	0 (0%)	13 (12.04%)	0 (0%)
Sinus arrhythmia	75 (32.89%)	1 (0.44%)	26 (24.07%)	0 (0%)
Diarrhea	67 (29.39%)	3 (1.32%)	14 (12.96%)	0 (0%)
Pharyngalgia	60 (26.32%)	0 (0%)	9 (8.33%)	0 (0%)
Proteinuria	58 (25.44%)	5 (2.19%)	12 (11.11%)	1 (0.93%)
Prolongation of the QT interval	57 (25%)	4 (1.75%)	15 (13.89%)	1 (0.93%)
GT elevation	55 (24.12%)	6 (2.63%)	16 (14.81%)	4 (3.7%)
Hyperglycemia	54 (23.68%)	0 (0%)	23 (21.3%)	1 (0.93%)
Oral mucositis	52 (22.81%)	2 (0.88%)	3 (2.78%)	0 (0%)
Hyperbilirubinemia	51 (22.37%)	2 (0.88%)	14 (12.96%)	1 (0.93%)
Hypothyroidism	47 (20.61%)	1 (0.44%)	4 (3.7%)	0 (0%)
Hoarseness	46 (20.18%)	0 (0%)	3 (2.78%)	1 (0.93%)
LDL elevation	40 (17.54%)	2 (0.88%)	7 (6.48%)	0 (0%)
Vomiting	39 (17.11%)	1 (0.44%)	10 (9.26%)	0 (0%)
Cough	37 (16.23%)	1 (0.44%)	14 (12.96%)	0 (0%)
Urine occult blood	35 (15.35%)	0 (0%)	8 (7.41%)	1 (0.93%)
Nausea	34 (14.91%)	0 (0%)	13 (12.04%)	0 (0%)
LYM reduction	34 (14.91%)	7 (3.07%)	12 (11.11%)	5 (4.63%)
ALT elevation	31 (13.6%)	0 (0%)	8 (7.41%)	0 (0%)
Hyponatremia	30 (13.16%)	7 (3.07%)	8 (7.41%)	3 (2.78%)
Abdominal pain	30 (13.16%)	1 (0.44%)	7 (6.48%)	0 (0%)
Hypoalbuminemia	28 (12.28%)	0 (0%)	12 (11.11%)	1 (0.93%)
AST elevation	28 (12.28%)	0 (0%)	8 (7.41%)	0 (0%)
ALP elevation	27 (11.84%)	4 (1.75%)	13 (12.04%)	2 (1.85%)
Dyspnea	27 (11.84%)	0 (0%)	8 (7.41%)	2 (1.85%)
Weight loss	27 (11.84%)	0 (0%)	2 (1.85%)	0 (0%)
Rash	25 (10.96%)	0 (0%)	6 (5.56%)	1 (0.93%)
Constipation	24 (10.53%)	0 (0%)	12 (11.11%)	0 (0%)
Hemoptysis	24 (10.53%)	3 (1.32%)	5 (4.63%)	1 (0.93%)
Dizziness	23 (10.09%)	0 (0%)	7 (6.48%)	0 (0%)
SCC	Anlotinib, n = 53		Placebo, n = 33	
Hypertension	30 (63.83%)	9 (19.15%)	5 (17.24%)	0 (0%)
Fatigue	23 (48.94%)	1 (2.13%)	2 (6.9%)	0 (0%)
TSH elevation	20 (42.55%)	0 (0%)	2 (6.9%)	0 (0%)
Anorexia	18 (38.3%)	0 (0%)	6 (20.69%)	0 (0%)
HFSR	16 (34.04%)	1 (2.13%)	3 (10.34%)	0 (0%)
Triglyceride elevation	15 (31.91%)	0 (0%)	5 (17.24%)	0 (0%)
Proteinuria	14 (29.79%)	2 (4.26%)	7 (24.14%)	0 (0%)
Cough	14 (29.79%)	0 (0%)	5 (17.24%)	0 (0%)
Hyperglycemia	13 (27.66%)	0 (0%)	8 (27.59%)	0 (0%)
Sinus arrhythmia	13 (27.66%)	0 (0%)	7 (24.14%)	0 (0%)
Diarrhea	13 (27.66%)	0 (0%)	3 (10.34%)	0 (0%)
TC elevation	13 (27.66%)	0 (0%)	3 (10.34%)	0 (0%)
Hemoptysis	13 (27.66%)	5 (10.64%)	2 (6.9%)	0 (0%)
Pharyngalgia	13 (27.66%)	1 (2.13%)	1 (3.45%)	0 (0%)
Prolongation of the QT interval	11 (23.4%)	3 (6.38%)	5 (17.24%)	1 (3.45%)
Hyperbilirubinemia	11 (23.4%)	0 (0%)	4 (13.79%)	1 (3.45%)
Hoarseness	11 (23.4%)	0 (0%)	3 (10.34%)	0 (0%)
LYM reduction	9 (19.15%)	2 (4.26%)	4 (13.79%)	0 (0%)
Hypophosphatemia	9 (19.15%)	1 (2.13%)	2 (6.9%)	0 (0%)
Oral mucositis	9 (19.15%)	1 (2.13%)	0 (0%)	0 (0%)
GT elevation	8 (17.02%)	1 (2.13%)	4 (13.79%)	2 (6.9%)
Hyponatremia	8 (17.02%)	5 (10.64%)	3 (10.34%)	1 (3.45%)
Vomiting	8 (17.02%)	0 (0%)	1 (3.45%)	0 (0%)
Hypothyroidism	8 (17.02%)	0 (0%)	0 (0%)	0 (0%)
Weight loss	8 (17.02%)	0 (0%)	0 (0%)	0 (0%)
Paresthesia	7 (14.89%)	0 (0%)	1 (3.45%)	0 (0%)
Urine occult blood	6 (12.77%)	0 (0%)	3 (10.34%)	0 (0%)
Rash	6 (12.77%)	0 (0%)	2 (6.9%)	0 (0%)
Nausea	6 (12.77%)	0 (0%)	1 (3.45%)	0 (0%)
Productive cough	6 (12.77%)	0 (0%)	1 (3.45%)	0 (0%)
LDL elevation	6 (12.77%)	0 (0%)	0 (0%)	0 (0%)
Dyspnea	5 (10.64%)	0 (0%)	4 (13.79%)	0 (0%)
Pectoralgia	5 (10.64%)	0 (0%)	1 (3.45%)	0 (0%)

Abbreviations: ACC, adenocarcinoma; ALP, alkaline phosphatase; ALT, alanine aminotransferase; AST, aspartate aminotransferase; GT, gamma transpeptidase; HFSR, hand and foot skin reaction; LDL, low‐density lipoprotein; LYM, lymphocyte; SCC, squamous cell carcinoma; TC, total cholesterol; TSH, thyroid‐stimulating hormone.

## 4 DISCUSSION

In this subgroup analysis, OS was significantly improved in ACC patients treated with anlotinib compared to ACC patients treated with placebo, but these findings were not observed in SCC patients. In the ACC subgroup, anlotinib significantly prolonged OS in patients with an EGFR mutation, an ECOG PS of 1, and >3 metastases and in patients who received two chemotherapy regimens and targeted regimens. OS was not improved in SCC patients treated with anlotinib, while PFS was significantly prolonged. The AEs observed in the SCC and ACC subgroups were similar.

In recent years, great progress has been made in the treatment of NSCLC, but the clinical outcome of patients with SCC is not as ideal as those with ACC. The DELTA trial assessed the effect of erlotinib in patients previously treated with one or two chemotherapies. There was no significant difference in outcomes between ACC patients treated with erlotinib and docetaxel. In patients with SCC, docetaxel is associated with prolonged PFS (HR: 1.60; 95% CI: 1.05‐2.43).^20^ The LUX‐Lung 8 trial compared the efficacy of afatinib and erlotinib in SCC patients who had progressed from previous platinum‐based chemotherapy. Afatinib significantly improved PFS (2.6 months vs 1.9 months, HR: 0.81, 95% CI: 0.69‐0.96, *P* = .010) and OS (7.9 months vs 6.8 months, HR: 0.81, 95% CI: 0.69‐0.95, *P* = .008) compared with erlotinib.^21^ However, erlotinib did not show superiority to docetaxel in SCC, and there was no head‐to‐head comparison between afatinib and docetaxel. Antiangiogenic therapies have been used in NSCLC treatment and showed benefit for non‐SCC patients. Bevacizumab, the first antiangiogenic agent against VEGF, shows the best treatment effect in addition to carboplatin‐paclitaxel chemotherapy, which improves OS (12.3 months vs 10.3 months, HR: 0.79; 95% CI: 0.67‐0.92).^22^ However, the benefits are modest and dependent on nonsquamous histology.^23^, ^24^, ^25^ These advances are observed mainly in non‐SCC patients, while there are few effective treatment options for SCC patients, except for necitumumab, which produces modest survival improvements in SCC patients.^26^ In this subgroup analysis, anlotinib significantly improved PFS not only in ACC patients (5.53 months vs 1.37 months; HR: 0.79; 95% CI: 4.40‐5.63) but also in SCC patients (5.63 months vs 2.7 months, HR: 0.79; 95% CI: 3.83‐7.00). OS was improved significantly in ACC patients (9.63 months vs 6.93 months; HR: 0.79; 95% CI: 8.17‐10.53), while it was prolonged without a statistically significant difference in SCC patients (10.7 months vs 6.00 months; HR: 0.79; 95% CI: 6.30‐15.13).

A previous meta‐analysis showed that the response to the first‐line regimen significantly impacted the efficacy of second‐line therapy.^27^ In addition to their ability to directly kill tumor cells, cytotoxic agents may also affect the immune response.^28^ The efficacy of nivolumab in NSCLC has been reported to be associated with the response to a first‐line chemotherapy regimen.^29^ Different resistance mechanisms will develop in patients with an EGFR mutation that progress from EGFR TKIs; for example, after treatment with gefitinib and erlotinib, the T790M mutation in exon 20,^30^, ^31^ amplifications in HER2 or mutations in MET, BRAF or phosphatidylinositol‐4,5‐bisphosphate 3‐kinase catalytic subunit alpha (PIK3CA), SCLC transformation will occur.^32^ Therefore, the effects of front‐line treatment for patients should also be considered in the subsequent treatment. This subgroup analysis showed that anlotinib prolonged PFS and OS in ACC patients who had received common chemotherapy regimens. In addition, patients previously treated with targeted agents also received an OS benefit.

In ALTER 0303 trial, many patients received subsequent treatment, especially in the placebo group. The proportions of ACC patients received subsequent chemotherapy and targeting‐drug therapy in the placebo group were more than that in the anlotinib group suggesting that the recorded OS benefit is attributable to anlotinib but not to either subsequent target or other therapies.

Anlotinib exhibited good tolerance, the AE profile observed in ACC patients and SCC patients were similar. More patients in the anlotinib group than in the placebo group discontinued treatment because of AEs, this could be associated with the longer duration of treatment in anlotinib group. Among the AEs induced by antiangiogenic drugs, major hemoptysis was strongly associated with patients with SCC histology.^33^ In the ALTER0303 trial, hemoptysis occurred in 27.66% of SCC patients treated with anlotinib, and the incidence of grade 3 or worse hemoptysis was 10.64%.

In conclusion, anlotinib improved survival in ACC patients treated with at least two lines of chemotherapy or a TKI. SCC patients who received anlotinib experienced a significant improvement in PFS and a tendency to experience prolonged OS. Anlotinib should be considered as an appropriate option for difficult‐to‐treat NSCLC patients as a subsequent treatment regardless of the histological type.

## CONFLICT OF INTERESTS

The authors have no conflict of interest to declare.

## AUTHOR CONTRIBUTIONS

YC was involved in the literature search, figures, study design, data collection, data analysis, and writing. BH, KL, QW, LZ, JS, ZW, JH, YS, WC, XW, YL, KN, FJ, BL, and JZ were involved in the article review.

## ETHICAL APPROVAL

This is a prespecified subgroup analysis in the ALTER0303 trial, and this study was approved by Jilin Cancer Hospital Ethics Committee on 22 June 2016.

## Data Availability

The data used to support the findings of this study are available from the corresponding author upon request.
