# Corrigendum

**DOI:** 10.1002/cam4.3701

**Published:** 2021-03-05

In the article by Ying Cheng et al., the authors have noticed that there is a mistake in Figure 1B (hazard ratio data) due to copying error and in Table 2 (SCC part) due to miscalculation error.

**TABLE 2:** Common adverse events (≥10%) in ACC and SCC patients

Adverse events	All grades	≥Grade 3	All grades	≥Grade 3
SCC	Anlotinib, n = 53		Placebo, n = 33	
Hypertension	34 (64.15%)	10 (18.87%)	7 (21.21%)	0 (0%)
Fatigue	25 (47.17%)	1 (1.89%)	5 (15.15%)	0 (0%)
TSH elevation	21 (39.62%)	0 (0%)	6 (18.18%)	0 (0%)
Anorexia	21 (39.62%)	0 (0%)	3 (9.09%)	0 (0%)
HFSR	19 (35.85%)	1 (1.89%)	3 (9.09%)	0 (0%)
Cough	16 (30.19%)	0 (0%)	4 (12.12%)	2 (6.06%)
TC elevation	15 (28.30%)	1 (1.89%)	3 (9.09%)	0 (0%)
Triglyceride elevation	15 (28.30%)	0 (0%)	4 (12.12%)	1 (3.03%)
Sinus arrhythmia	15 (28.30%)	0 (0%)	3 (9.09%)	0 (0%)
Diarrhea	15 (28.30%)	0 (0%)	2 (6.06%)	0 (0%)
Hemoptysis	14 (26.42%)	5 (9.43%)	3 (9.09%)	1 (3.03%)
Proteinuria	14 (26.42%)	2 (3.77%)	7 (21.21%)	0 (0%)
Pharyngalgia	14 (26.42%)	1 (1.89%)	8 (24.24%)	0 (0%)
Hoarse	14 (26.42%)	1 (1.89%)	2 (6.06%)	0 (0%)
Hyperglycemia	13 (24.53%)	0 (0%)	0 (0%)	0 (0%)
Hyperbilirubinemia	13 (24.53%)	0 (0%)	0 (0%)	0 (0%)
Prolongation of the QT interval	11 (20.75%)	3 (5.66%)	0 (0%)	0 (0%)
Hypophosphatemia	11 (20.75%)	1 (1.89%)	4 (12.12%)	0 (0%)
Oral mucositis	11 (20.75%)	1 (1.89%)	1 (3.03%)	0 (0%)
Hyponatremia	10 (18.87%)	6 (11.32%)	1 (3.03%)	0 (0%)
GT elevation	10 (18.87%)	2 (3.77%)	1 (3.03%)	0 (0%)
Weight loss	10 (18.87%)	0 (0%)	1 (3.03%)	0 (0%)
LYM reduction	9 (16.98%)	2 (3.77%)	5 (15.15%)	1 (3.03%)
Nausea	9 (16.98%)	0 (0%)	7 (21.21%)	0 (0%)
LDL elevation	8 (15.09%)	0 (0%)	9 (27.27%)	0 (0%)
Vomiting	8 (15.09%)	0 (0%)	6 (18.18%)	0 (0%)
Rash	8 (15.09%)	0 (0%)	3 (9.09%)	0 (0%)
Hypothyroidism	8 (15.09%)	0 (0%)	2 (6.06%)	0 (0%)
Urine occult blood	7 (13.21%)	0 (0%)	3 (9.09%)	0 (0%)
Paresthesia	7 (13.21%)	0 (0%)	2 (6.06%)	0 (0%)
Dyspnea	7 (13.21%)	0 (0%)	1 (3.03%)	0 (0%)
Productive cough	6 (11.32%)	0 (0%)	0 (0%)	0 (0%)

The authors have mistakenly put the incorrect values in Figure 1B (hazard ratio data) and also the relevant values are revised in the section under Discussion in text part. The correct version of Figure 1B is displayed below:

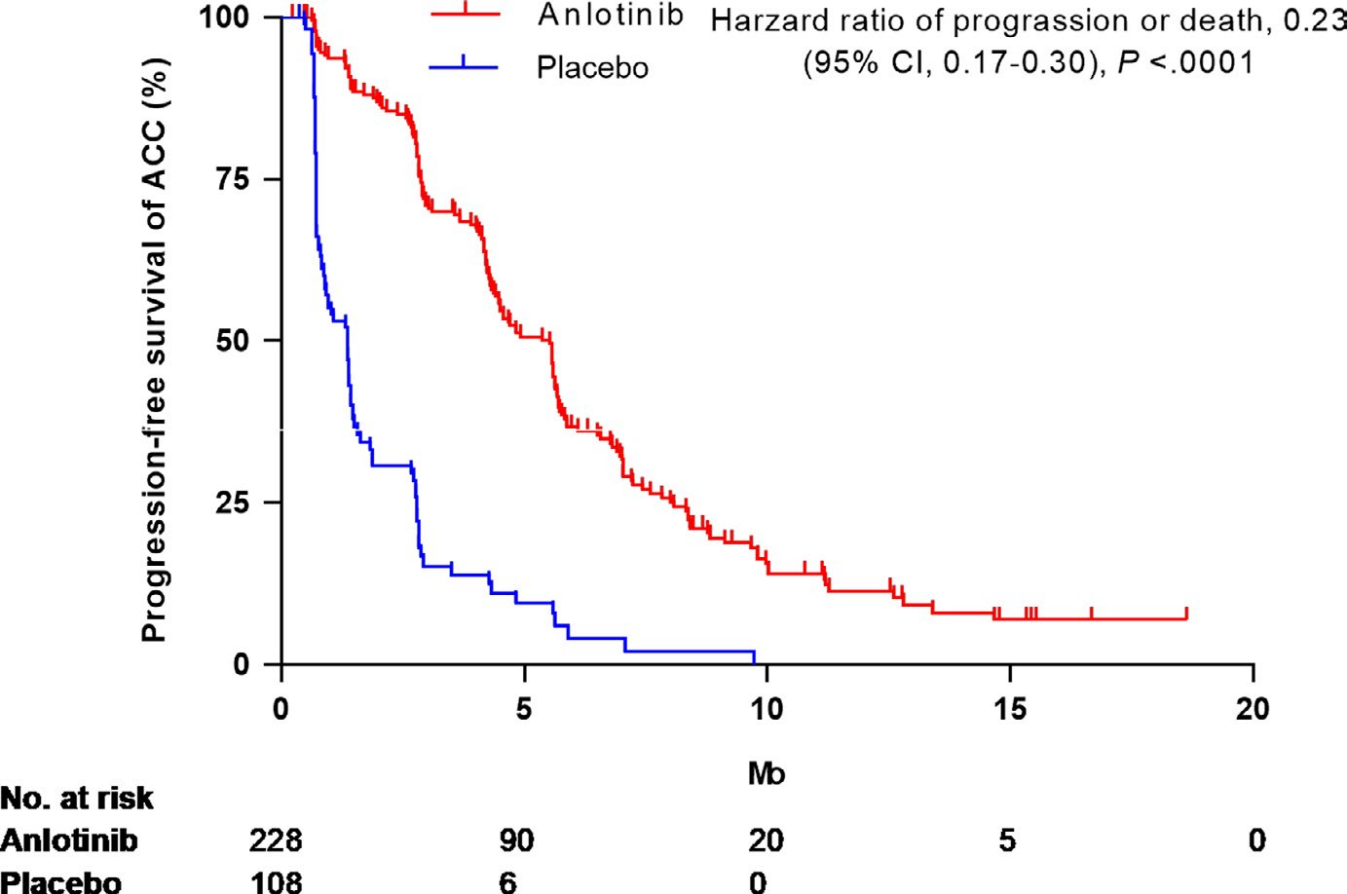

Revised relevant values in the text are as the followings:

‐DISCUSSION (page 2626, left column, line 4)

In this subgroup analysis, anlotinib significantly improved PFS not only in ACC patients (5.5 months vs 1.4 months; HR: 0.23; 95% CI: 0.17–0.30) but also in SCC patients (4.8 months vs 2.7 months, HR: 0.43; 95% CI: 0.18–0.59). OS was improved significantly in ACC patients (9.6 months vs 6.9 months; HR: 0.68; 95% CI: 0.48–0.88), while it was prolonged without a statistically significant difference in SCC patients (10.7 months vs 6.5 months; HR: 0.75; 95% CI: 0.43–1.25).

The authors have miscalculated in Table 2 (SCC part) and also relevant values are revised in the section under Discussion. The correct version of Table 2 is displayed below:

Revised relevant values in the text are as the followings:

DISCUSSION (page 2628, left column, line 6)

In the ALTER0303 trial, hemoptysis occurred in 26.42% of SCC patients treated with anlotinib and the incidence of grade 3 or worse hemoptysis was 9.43%.

The authors have made changes in text for Figure 2A,B citation in text.

A figure number quoting mistake revised as the followings:

‐RESULTS 3.3 | Subgroup analysis in ACC and SCC (page 2624, right column)

In ACC patients, anlotinib significantly prolonged PFS in most subgroups (Figure 2B).

OS was significantly prolonged with anlotinib in patients with an EGFR mutation, an Eastern Cooperative Oncology Group performance status (ECOG PS) score of 1, and >3 metastases and in patients who received two chemotherapy regimens or targeted regimens (Figure 2A).

The authors apologize for these errors.
