# Supplementary material for: Effect of anlotinib as a third‐ or further‐line therapy in advanced non‐small cell lung cancer patients with different histologic types: Subgroup analysis in the ALTER0303 trial
Source: Cancer Med. 2020 Feb 16;9(8):2621–30. doi: 10.1002/cam4.2913 (PMC7163095; doi:10.1002/cam4.2913)
Supplement: Supplementary file 1 [file CAM4-9-2621-s001.docx]

**Supplementary table 1.** Tumor Response with anlotinib versus placebo for ACC and SCC subtypes.

|  | **ACC, N=336** | | **SCC, N=86** | |
| --- | --- | --- | --- | --- |
|  | Placebo, n=108 | Anlotinib, n=228 | Placebo, n=33 | Anlotinib, n=53 |
| **Partial response** | 1 (0.93%) | 22 (9.65%) | 0 | 4 (7.55%) |
| **SD decreases** | 7 (6.48%) | 102 (44.74%) | 5 (15.15%) | 21(39.62%) |
| **SD increases** | 28 (25.93%) | 65 (28.51%) | 12 (36.36%) | 13 (24.53%) |
| **Progressive disease** | 61 (56.48%) | 21 (9.21%) | 11 (33.33%) | 11 (20.75%) |
| **NE** | 11 (10.19%) | 18 (7.89%) | 5 (15.15%) | 4 (7.55%) |
| **ORR (CR+PR, %)^a^** | 1 (0.93%) | 22 (9.65%) | 0 | 4 (7.55%) |
| **DCR (CR+PR+SD, %)^a^** | 36 (33.33%) | 189 (82.89%) | 17 (51.51%) | 38 (71.70%) |

Abbreviations：NE= not evaluate; DCR=disease control rate; ORR=overall response rate;

^a^ There was a statistical difference between the two groups of ACC subgroups (two-sided test). P=0.002 for the ORR; P<0.0001 for the DCR.

^b^ There was a statistical difference between the two groups of SCC subgroups (two-sided test). P=0.2758 for the ORR; P=0.0580 for the DCR.

**Supplemental Table** **2.** Subsequent treatment of patients with adenocarcinoma and squamous cell carcinoma.

| **Type of treatment** | **Adenocarcinoma** | | **Squamous cell carcinoma** | |
| --- | --- | --- | --- | --- |
|  | Anlotinib group  N=228 | Placebo group  N=108 | Anlotinib group  N=53 | Placebo group  N=33 |
| **Chemotherapy** | 66 (28.95%) | 43 (39.81%) | 17 (32.08%) | 6 (18.18%) |
| **Targeting-drug therapy** | 48 (21.05%) | 26 (24.07%) | 12 (22.64%) | 8 (24.24%) |
| **Radiotherapy** | 18 (7.90%) | 4 (3.70%) | 4 (7.55%) | 2 (6.06%) |
| **Traditional Chinese medicine** | 28 (12.28%) | 10 (9.26%) | 6 (11.32%) | 7 (21.21%) |
| **Surgery** | 7 (3.07) | 1 (0.93%) | 1 (1.89%) | 0 |
| **Others or missing data** | 61 (26.75%) | 24 (22.22%) | 13 (24.53%) | 10 (30.30%) |
